# T-Lymphocyte Subsets in Apparently Healthy Nigerian Children

**DOI:** 10.1155/2010/474380

**Published:** 2010-02-11

**Authors:** Emmanuel Oni Idigbe, Rosemary A. Audu, Edna O. Iroha, Adebola O. Akinsulie, Edamisan Olusoji Temiye, Veronica C. Ezeaka, Ifedayo M. O. Adetifa, Adesola Z. Musa, Joseph Onyewuche, Sylvester U. Ikondu

**Affiliations:** ^1^Human Virology Laboratory, Nigerian Institute of Medical Research, Yaba, Lagos, Nigeria; ^2^The Paediatrics HIV/AIDS Working Group, Department of Paediatrics, Lagos University Teaching Hospital, Idi-Araba, Lagos, Nigeria

## Abstract

Population studies showed that there are differences in T-lymphocytes subpopulation of normal children in different regions, and reference values in an area might be different from another. This study compared the values in our population with CDC and WHO reference values. Blood samples from 279 healthy, HIV-negative children <12 years of age were analysed for complete blood count, CD3+, CD4+, CD8+ counts and percentages. Except for CD8%, mean values for all parameters measured significantly decreased with age. CD4+ counts were higher in females than males, *P* < .05. Using the WHO criteria, 15.9% of subjects had low total lymphocyte count and 20.6% had low CD4 count. Children <3 years had median CD4% lower than WHO normal values. Our median CD4+ counts correlated with CDC values. Values used by WHO in infants are higher than ours. We suggest that our children be assessed using CDC reference values which correlate with ours.

## 1. Introduction

The mature T-lymphocytes are defined by the presence of CD3, and either CD4+ or CD8+ unit antigens [1]. The CD4+ antigen-bearing T-lymphocyte subset also known as the T-helper cells has become popular since the advent of the Human Immunodeficiency Virus (HIV) infection. These T-lymphocytes are the primary targets of the HIV infection because the CD4+ antigen is the primary binding site of the HIV [2–4]. CD4+T cells serve as both essential regulators and effectors of the immune response; infection with HIV induces a progressive loss of these cells [5]. Profound decline of these cells underlies the immunodeficiency that results in Acquired Immunodeficiency Syndrome (AIDS) [2]. The CD4+ T-cell count is the standard for assessing the immunologic progression of the disease, determination of need to commence antiretroviral (ARV) treatment, chemoprophylaxis for opportunistic infections, and for monitoring or modifying antiretroviral treatment [6, 7]. 

An adult individual has about 3000 lymphocytes per mm [3] in the peripheral blood with 70%–80% being T-lymphocytes and 65% of these T-lymphocytes bear CD4+ antigens [8, 9]. In children, the number of circulating T-cells increases from mid-gestation until the infant is about 6 months. This peak is followed by a gradual decline throughout childhood until adult levels are reached by late childhood [10]. As a result of the age-related changes in the absolute lymphocyte numbers and thus CD4+ count, the Centers for Disease Control and Prevention (CDC) in classifying children into immune categories using CD4+, produced a system based on specific age groups [11]. The World Health Organization (WHO) has also evolved an age specific CD4+ system for use in children [12]. In Caucasian, Asian, and some African populations, studies have been done to obtain reference values of CD4+ counts in the normal population and from these centile charts absolute counts have been published. These studies are not uniform in their reports of reference CD4+ counts in normal children [13–15]. Anglaret et al. in 1997 found that CD4+ absolute counts were one-third higher in HIV- infected adults in francophone West Africa compared to their French counterparts [14]. Njoku et al. in a similar study in Jos, Nigeria also confirmed this report in Nigerian adults [16]. However another study done in Lagos showed that Nigerian adults had lower CD4+ counts compared to the Caucasians [17].

It has been our experience in the Paediatrics HIV clinic at Lagos University Teaching Hospital (LUTH) that most of the HIV-infected infants and older children we managed had profound CD4+ lymphocytopenia. While some studies had been done to determine normal lymphocyte subpopulations in the Nigerian adults [16–18] the authors are not aware of any published work that has established a reference value for CD4+ count in Nigerian children. In this study we sought to evaluate the normal absolute lymphocyte, CD4+ and CD8 counts in apparently healthy Nigerian children; determine the occurrence or otherwise of non-HIV-related CD4+ lymphocytopenia; compare values obtained with age-related values of CDC and WHO. Finally we attempted to correlate the absolute lymphocyte counts with CD4+ counts.

## 2. Subjects and Methods

The study was carried out at the well-baby and Community Health outpatient clinics of LUTH, Idi-Araba, and a private nursery and primary school located about 2 kilometers away. The subjects were children aged 12 years and below who were apparently well at least 2 weeks before recruitment and were not on any medication except multivitamins, or were healthy term neonates born to HIV-negative healthy mothers and weighed at least 2500 g at birth. Excluded were children who were known to have sickle cell anaemia, HIV-exposed, HIV positive, or who had been on steroids, antituberculosis drugs, and systemic antifungal agents. Also excluded were children who were obviously clinically ill including those having a febrile illness or recovered from such less than 2 weeks before recruitment; or neonates who had suffered from intrauterine growth retardation. 

Approval for the study was obtained from the hospital's Ethical Committee and written informed consent was obtained from the parents/caregivers. History concerning the child's health, haemoglobin genotype, the last episode of febrile illness, immunizations and drugs administered to the child in the preceding two weeks, mother's health, and baby's condition at birth was obtained at time of recruitment. Demographic data including age, sex, height, and weight were also obtained. 

Three hundred children were randomly selected at the study centers and stratified equally into four age groups: 0–28 days, 29 days–1year, >1–6 years, and >6–12 years. Three to four milliliters of venous blood was taken from each of the eligible subjects using potassium ethylene diamine tetra-acetic acid (EDTA) bottles daily on Mondays to Fridays between the hours of 10.00 AM and 12 noon and were transported to the Human Virology Laboratory of the Nigerian Institute of Medical Research (NIMR), Yaba, where analyses were done within 4 hours of arrival at the laboratory. The distance between the places of collection was not more than 4 km, and it takes an average of 30 minutes to deliver the samples to the laboratory.

The blood samples obtained were screened for HIV antibody using HIV antibody ELISA and Western blot electrophoresis. Thereafter all the samples had a complete blood count done using an automated haemoanalyzer. The following variables were determined: the total white blood cell count (TWC) and differential white blood cells including total lymphocyte count (TLC) and percentage lymphocyte, the packed cell volume and haemoglobin concentration. The CD3, CD4+, and CD8 counts and CD4 /CD8 ratio were done on the same blood sample using FACScount) (Becton-Dickinson Immunocytometry Systems, San Jose, CA, USA) at the same Virology laboratory of NIMR, Yaba. The CD4 percentage was then determined by dividing the CD4 count by the TLC multiplied by 100.

Data generated were entered into SPSS version 11. The values obtained were then tabulated by age groups and sex. Data analysis of frequencies, means, standard deviations, correlation, and comparisons were done. Student's *t*-test and one-way analysis of variance were employed to determine the relationship between the different subpopulations. The 5th, 50th, and 95th percentile values were also determined and the 50th percentile values were compared with set values as determined by CDC [11] and WHO [12] criteria and the figures were generated using Microsoft excel. The *P*-values were taken to be significant when less than .05.

## 3. Results

Out of the 300 samples collected, 21 (7.0%) were excluded from analysis because of laboratory errors or faulty sample collection. Of the remaining 279 subjects, 147 (52.7%) were males and 132 (47.3%) females. The mean age of the subjects was 37.8 ± 41.4 months; the mean age for males was 38.3 ± 41.7 months; that of the females was 37.8 ± 41.5 months. The difference in the age between the sexes was not statistically significant (*t* = 0.21 *d*f = 277; *P* > .05). All the mothers of the 61 neonates included in the study were found to be HIV negative. Also, all the subjects, including the neonates, were found to be HIV negative.

The FACScount machine used in this study could not determine CD4 count above 2000 cells/*μ*L. The CD4 count in 45 children was greater than 2000 cells/*μ*L; 39 of whom was subjects less than one year old, 4 were between 1 and 5 years, and the remaining 2 were in the age group >6–12 years. However these results were all recorded as 2000 cells/*μ*L. 

Table 1 showed the results of total white cell count (TWC), total lymphocyte counts (TLC) and percentages, CD3, CD4, and CD8 counts and percentages, and CD4 : CD8 in the different age groups. The mean values significantly decreased with increasing age for TWC, TLC, CD3, and CD4 counts, CD4% and CD4 : CD8 ratio. When all the subjects were considered, there was no statistical difference in these variables between the males and the females, except in the CD4 counts where the value was significantly higher in females than the males (1.40 ×10^9^ versus 1.26 ×10^9^; *t* = −2.44, *P* < .05). Also, when segregated into different age groups there were no statistically significant differences in the values of the measured variables between males and females except in the age group 6–12 years where the mean total lymphocyte count (2.96 versus 3.43 ×10^9^; *t* = −2.04, *P* < .05), CD4 cell count (0.95 versus 1.25 ×10^9^; *t* = −3.28, *P* < .05), CD4 percentage (31.90 versus 36.17; *t* = −2.21, *P* < .05) and the CD4 : CD8 ratio (1.46 versus 2.06; *t* = −4.43, *P* < .001) were significantly higher in the females (Table 2). The total lymphocyte count was also correlated with the CD4 counts, adjusting for the age of the subjects. There was high correlation between the total lymphocyte count and CD4 count in the study, *r* = 0.65, *P* < .001

The subjects were further rearranged into the WHO and CDC age group and set-values for TLC, CD4 count, and CD4 percentages. Using the WHO criteria for TLC, 43 (15.9%) had values considered to be low, 30 (69.8%) of whom were less than 1 year. For CD4 count, 54 (20.6 %) had CD4 in the range considered to be low. Fifty-one (94.4%) of these were less than one year of age and the remaining 3 (5.6%) were in the age group 12–35 months. For CD4 percentage, 55.9% were normal while 12.5% were in the very low range (Table 3). 

When compared with CDC set values, 172 (65.6%) had CD4 count in the normal range while 5 (2.0%) had values considered to be very low. Four of these five were less than 1 year of age. Also 188 (74%) had CD4 percent values considered normal. Only 3 (1.2 %) had values regarded to be very low. These 3 were in the age range 1–5 years (Table 4). 

The WHO and CDC age determined CD4 count and CD4 percent were compared with the 50th percentiles for age in this study. Since WHO CD4 percent for children older than 5 years was not stated but was expressed as CD4 count the value of 25% was used for children older than 5 years for purpose of comparison.

**Figure 1:**
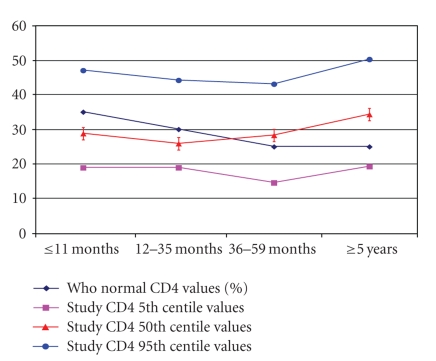
Comparison of median CD4 percent with the WHO normal values.

The 50th percentile CD4 percent by age in this study was found to be significantly lower than the set values by WHO in the age group ≤11 months (35% for WHO versus 28.8% in our study) and 12–35 months (30% WHO versus 25.9% in our study), respectively. However the values were higher in the age groups 36–59 months (25% WHO versus 28.3% in the study) and ≥5 years (25% WHO versus 34.3% in the study) (Figure 1). Conversely the comparisons of the 50th percentile for CD4 percent in this study were found to be significantly higher than the set points for CDC in all age groups (28.8%, 28.3%, and 33.4% for ages <1yr, 1–5 yrs, 6 yrs, above, resp.).

**Figure 2:**
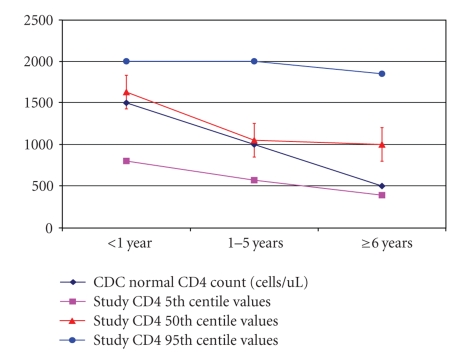
Comparison of the median CD4+ count by age with normal CDC counts.

The closest comparison was however in the CD4 count values of CDC and the 50th percentile values in our study where there was no statistical differences in study values and CDC values in the age groups <1 year (1,500 CDC versus 1,624 cells/*μ*L for our study) and those between 1 and 5 years 1,000 cells/*μ*L CDC versus 1,047 cells/*μ*L for our study), but the value was much higher in the age group 6 years and above (500 cells/*μ*L CDC versus 996 cells/*μ*L for our study) (Figure 2).

However, five children, 4 of whom were less than one year and the remaining in the age range 1–5 years, had CD4 count values less than 750 cells/*μ*L and 500 cells/*μ*L respectively, considered to be severe immunosuppression levels (Table 4).

## 4. Discussion

This study showed that there is a wide variation between the 5th percentile and the 95th percentile in the total white cell, the lymphocyte counts, and its subsets in the children studied. The variation is the widest in neonates and the infants. Also there is a steady decline in the median counts in the total white cell count, total Lymphocyte count, and CD4 counts with age. These findings are in conformity with the previous reports of haematological parameters in children [10, 19–21]. It was observed that the CD4 cell count was significantly higher in the females than males in our study. This observation was largely due to the higher levels of CD4 cells in female children older than 5 years. This is in agreement with some studies in Asian and European children [19, 22–24]. However several studies in the Africans showed that there was no significant gender difference in the CD4 count of children until adolescence, after which females tend to have higher CD4 count than males [25–27]. The reasons for this disparity is not clear but both genetic and environmental factors may play a role. 

The median CD4 percentage in this study ranged from 35.9% in the neonates to the lowest level of 26.6% in infants, and then gradually rose to 33.8% at 6–12 years. These values were lower than those reported in the developed countries [10, 22, 26] but were in agreement with findings in other African Children [19, 27, 28]. This observed differences, which had been found consistently in African children, are of importance when CD4 percentage is to be used to determine commencement of prophylaxis and antiretroviral therapy in HIV-infected children. One study conducted in West Africa observed that a CD4 percentage of 25%, with clinical staging, provided a reliable threshold for diagnosing severe immunosuppression and commencement of antiretroviral therapy in infants less than 6 months of age [29]. It is salutary to note that in most of the studies referred to above more than 95 percent of the children have CD4 percentage greater than 25%. Therefore, in the setting where virologic diagnosis of HIV in infants less than 1 year of age is not available, the threshold of CD4 percent of less than 25% could be a useful tool for the commencement of ARV. 

Since CD4 count or CD4% are important determinants for commencing an HIV-infected child on antiretroviral treatment, cut-off values for the commencement of therapy have been determined by WHO [12] and CDC [11], and these two organizations have important different cut-off values. We therefore used these values to compare the total lymphocyte count, CD4 counts, and percentages obtained in this group of normal, HIV-uninfected children. Using the WHO criteria, 54 (20.6%) of the children would have been considered to have severe immune deficiency requiring the commencement of antiretroviral therapy if they had been HIV positive; see Tables 6 and 7. All the affected children were less than 3 years of age with majority falling below one year. Similar scenario exists for the use of total lymphocyte count and percentage CD4 count. Indeed, 24.8% of children less than 1 year would have been classified as having severe immunodeficiency if they were HIV positive using the WHO criteria. 

The number of children who would have been classified as having deficient levels of CD4 count was expectedly less with CDC criteria. In this study therefore, it would seem that the criteria used for setting the WHO cut-off points were rather too high. Most of the studies used by WHO in arriving at its cut-off points were derived from developed countries. Several studies in Africa however showed that HIV-uninfected African Children have lower CD4 count and percentages than their European and American counterparts [19, 27, 28]. Therefore, it might be necessary for WHO to review its cut-off points to reflect these differences. 

It has been suggested that the CD4 : CD8 ratio could be a useful diagnostic tool in infants less than 18 months of age where virologic testing is unavailable [30, 31]. In healthy children, the CD8 count is about 30% of total lymphocyte count while the CD4 count is 60% [30]. This usually gives a CD4 : CD8 ratio of greater than one. In the current study, however, the median CD8 count was 17.2% (range 13.7% in infants 29 days to less than one year to 20.7% in children one year and above) of total lymphocyte count. The low CD8% found in this study is also in keeping with the generally low CD8 percent found in some African studies with lower values found in infants than in older age groups [27, 28]. The median CD4 : CD8 ratio ranged from 2.36 in the neonatal period and decreasing gradually to 1.68 in the age group 6–12 years. This is in agreement with previous studies in Africa and other regions [24–28]. As infection with HIV destroys the CD4 cells and the immunologic system attempts to eliminate the virus, the CD8 cells rise and CD4 cells decrease. A study done in Zimbabwe showed that a CD4 : CD8 ratio of less than one was highly predictive of HIV infection; CD4 : CD8 ratio was found to have greater than 98% sensitivity and greater than 98% specificity in identifying HIV-infected infants [30]. The technique was found to be more cost-effective and faster than the standard virologic testing. Reversal of CD4 : CD8 ratio in our HIV exposed children could therefore be a useful diagnostic tool in predicting HIV infection in HIV-exposed children who are less than 18 months old. However, CD8 count is not routinely done in many resource-limited countries including our centre. 

In conclusion, these findings suggest that some of the criteria used by WHO are rather high when compared to the values obtained in our normal children. The presence of low CD4 count in these subjects suggests that idiopathic low CD4 count may occur in children in our environment. This may explain why some children who were HIV positive but with low CD4 count remain in good health and might not be functionally immunodeficient. It is however, difficult to identify such individuals in the face of HIV infection since their premorbid CD4 count or percentage values would not be available for comparison. However since the median values in this study correlate well with the set values, especially with the CDC, we would suggest that our children should still be assessed using these values; especially as the recent studies are strongly pointing towards starting ART at higher CD4 count levels for optimal benefit in other to reduce morbidity and mortality in HIV infected individuals.

## Figures and Tables

**Table 1:** Total white cell count, total lymphocytes count, subsets, and percentages for healthy children by age group.

Phenotype (cells ×10^3^)	Age group				Total
	0–28 days	29 days–<1 years	1–5 years	6–12 years	
TWCC	n* = 61 (22.6)	*n* = 62 (23.0)	*n* = 74 (27.4)	*n* = 73 (27.0)	*n* = 270 (100.0)
Mean (±SD)	11.16 (3.60)	9.25 (3.18)	7.85 (2.26)	6.39 (2.13)	8.53 (3.29)
50th (5th, 95th) centiles	10.70 (5.73, 17.38)	8.95 (4.50, 16.01)	7.65 (4.75, 12.00)	6.20 (2.50, 10.43)	8.10 (4.36, 14.39)

TLC	*n* = 61 (22.6)	*n* = 62 (23.0)	*n* = 74 (27.4)	*n* = 73 (27.0)	*n* = 270 (100.0)
Mean (±SD)	5.08 (2.02)	6.26 (2.28)	4.06 (1.34)	3.17 (1.05)	4.56 (2.04)
50th (5th, 95th) centiles	4.85 (2.52, 8.70)	6.10 (2.88, 10.75)	3.95 (2.20, 6.15)	3.10 (1.40, 4.90)	4.10 (2.20, 8.65)

% lymphocyte count	*n* = 61 (22.6)	*n* = 62 (23.0)	*n* = 74 (27.4)	*n* = 73 (27.0)	*n* = 270 (100.0)
Mean (±SD)	47.23 (15.05)	67.61 (8.21)	51.90 (8.18)	50.76 (10.10)	54.10 (12.98)
50th (5th, 95th) centiles	46.40 (25.08, 69.88)	67.7 (55.24, 82.14)	51.90 (37.08, 64.75)	50.00 (33.81, 70.46)	54.10 (31.32, 74.88)

CD3	*n* = 57 (21.8)	*n* = 62 (23.7)	*n* = 74 (28.2)	*n* = 69 (26.3)	*n* = 262 (100.0)
Mean (±SD)	2.59 (0.73)	2.44 (0.81)	2.19 (0.69)	1.94 (0.66)	2.27 (0.76)
50th (5th, 95th) centiles	2.50 (1.44, 3.50)	2.37 (0.98, 3.50)	2.18 (1.14, 3.49)	1.85 (0.79, 3.32)	2.21 (1.04, 3.50)

CD4	*n* = 57 (21.8)	*n* = 62 (23.7)	*n* = 74 (28.2)	*n* = 69 (26.3)	*n* = 262 (100.0)
Mean (±SD)	1.66 (0.35)	1.49 (0.44)	1.16 (0.43)	1.08 (0.42)	1.33 (0.47)
50th (5th, 95th) centiles	1.76 (1.11, 2.00)	1.53 (0.64, 2.00)	1.05 (0.57, 2.00)	1.00 (0.39, 1.85)	1.28 (0.58, 2.00)

CD4 percentage	*n* = 55 (21.7)	*n* = 58 (22.8)	*n* = 74 (29.1)	*n* = 67 (26.4)	*n* = 254 (100.0)
Mean (±SD)	35.93 (9.03)	26.58 (6.23)	29.19 (8.17)	33.81 (8.10)	31.27 (8.67)
50th (5th, 95th) centiles	35.71(22.65, 50.14)	26.06 (18.50, 43.94)	28.26 (17.27, 44.95)	34.33 (19.47, 48.78)	30.09 (18.96, 46.66)

CD8 count	*n* = 57 (21.8)	*n* = 62 (23.7)	*n* = 74 (28.2)	*n* = 69 (26.3)	*n* = 262 (100.0)
Mean (±SD)	0.74 (0.30)	0.80 (0.42)	0.84 (0.38)	0.67 (0.31)	0.76 (0.36)
50th (5th, 95th) centiles	0.72 (0.32, 1.23)	0.72 (0.21, 1.61)	0.77 (0.35, 1.61)	0.63 (0.19, 1.44)	0.69 (0.32, 1.57)

CD8 Percent	*n* = 55 (21.7)	*n* = 58 (22.8)	*n* = 74 (29.1)	*n* = 67 (26.4)	*n* = 254 (100.0)
Mean (±SD)	15.07 (4.51)	13.47 (4.37)	20.74 (6.51)	20.99 (6.60)	17.92 (6.58)
50th (5th, 95th) centiles	14.32 (9.20, 24.35)	13.67 (7.22, 21.05)	20.74 (10.17, 30.25)	20.70 (11.87, 30.32)	17.20 (8.70, 29.25)

CD4 : CD8	*n* = 57 (21.8)	*n* = 62 (23.7)	*n* = 74 (28.2)	*n* = 69 (26.3)	*n* = 262 (100.0)
Mean (±SD)	2.54 (1.06)	2.24 (1.15)	1.57 (0.70)	1.72 (0.62)	1.98 (0.97)
50th (5th, 95th) centiles	2.36 (1.33, 4.18)	2.10 (1.07, 3.51)	1.49 (0.69, 2.89)	1.68 (0.82, 2.89)	1.81 (0.84, 3.51)

Note: n* Subjects with missing values were excluded, TWCC: Total White Cell Count, TLC: Total Lymphocyte Count.

**Table 2:** Distribution of T-lymphocyte subsets by gender and age group.

Lymphocyte subsets	Age groups by gender							
	0–28 days		29 days–<1 year		1–5 years		6–12 years	
	Male	Female	Male	Female	Male	Female	Male	Female
TLC (*μ*L ×10^9^)								
Number (%)	31 (11.5)	30 (11.1)	30 (11.1)	32 (11.9)	38 (14.1)	36 (13.3)	40 (14.8)	33 (12.2)
Mean ± SD	5.26 (2.25)	4.95 (1.76)	5.78 (2.23)	6.71 (2.27)	4.24 (1.29)	3.88 (1.38)	2.96 (0.91)	3.43 (1.07)
Median	4.90	4.55	5.40	6.20	4.35	3.70	2.85	3.40

CD4 count (*μ*L ×10^9^)								
Number (%)	30 (11.5)	27 (10.3)	31 (11.8)	31 (11.8)	38 (14.5)	36 (13.7)	39 (14.9)	30 (11.5)
Mean ± SD	1.64 (0.37)	1.69 (0.33)	1.41 (0.47)	1.57 (0.40)	1.17 (0.39)	1.16 (0.48)	0.95 (0.38)	1.25 (0.40)
Median	1.66	1.78	1.33	1.62	1.06	1.02	0.89	1.16

CD4%								
Number (%)	28 (11.0)	27 (10.6)	28 (11.0)	30 (11.8)	38 (15.0)	36 (14.2)	37 (14.6)	30 (11.8)
Mean ± SD	35.08 (8.88)	36.80 (9.27)	27.48 (6.55)	25.75 (5.90)	28.30 (7.24)	30.13 (9.06)	31.90 (9.06)	36.17 (6.04)
Median	35.42	35.71	26.41	24.88	28.05	28.58	32.96	36.71

CD8 count (*μ*L ×10^9^)								
Number (%)	30 (11.5)	27 (10.3)	31 (11.8)	31 (11.8)	38 (14.5)	36 (13.7)	39 (14.9)	30 (11.5)
Mean ± SD	0.73 (0.36)	0.76 (0.21)	0.79 (0.43)	0.81 (0.42)	0.88 (0.41)	0.79 (0.34)	0.69 (0.36)	0.65 (0.24)
Median	0.65	0.75	0.71	0.74	0.80	0.72	0.65	0.62

CD8%								
Number (%)	28 (11.0)	27 (10.6)	28 (11.0)	30 (11.8)	38(15.0)	36 (14.2)	37 (14.6)	30 (11.8)
Mean ± SD	13.76 (3.71)	16.44 (4.90)	14.40 (4.54)	12.60 (4.08)	20.75 (6.36)	20.74 (6.74)	22.98 (7.46)	18.54 (4.35)
Median	13.53	15.34	15.47	12.04	20.33	20.54	22.48	18.72

CD4 : CD8 Ratio								
Number (%)	30 (11.5)	27 (10.3)	31 (11.8)	31 (11.8)	38 (14.5)	36 (13.7)	39 (14.9)	30 (11.5)
Mean ± SD	2.70 (1.28)	2.37 (0.74)	2.20 (1.42)	2.28 (0.82)	1.53 (0.71)	1.62 (0.71)	1.46 (0.52)	2.06 (0.59)
Median	2.54	2.16	2.00	2.25	1.44	1.53	1.42	2.05

**Table 3:** Distribution of total lymphocyte count, CD4 count, and percentage using WHO cut-off values.

Total lymphocyte count and CD4 groupings	Age group								Total *n* (%)
	≤11 months		12–35 months		36–59 months		≥5 years		
	WHO	Study *n* (%)	WHO	Study *n* (%)	WHO	Study *n* (%)	WHO	Study *n* (%)	
CD4 count (cells/ul) *n* = 262	<1,500	51 (42.9)	<750	3 (10.0)	<350	0	<200	0	54 (20.6)
TLC (cells/ul) *n* = 270	<4000 *n* = 123	30 (24.4)	<3000 *n* = 30	4 (13.3)	<2500 *n* = 33	3 (9.1)	<2000 *n* = 84	6 (7.1)	43 (15.9)

WHO CD4 %	≥35	34 (30.1)	≥30	12 (40.0)	≥25	21 (63.6)	≥500	76 (95.0)	143 (55.9)
	30–34.9	15 (13.3)	25–29.9	4 (13.3)	20–24.9	9 (27.3)	350–499	3 (3.8)	31 (12.1)
	25–29.9	36 (31.8)	20–24.9	12 (40.0)	15–19.9	1 (3.0)	200–349	1 (1.2)	50 (19.5)
	<25	28 (24.8)	<20	2 (6.7)	<15	2 (6.1)	<200 or <15%	0	32 (12.5)

**Table 4:** Distribution of CD4 count by age group in study subject using CDC cut-off values.

	Age group						Total *n* (%)
	<12 months		12–71 months		≥6 years		
CDC classification (cells/*μ*L)	CDC	Study *n* (%)	CDC	Study *n* (%)	CDC	Study *n* (%)	
	≥1,500	68 (57.1)	≥1,000	39 (52.7)	>500	65 (94.2)	172 (65.6)
	750–1,499	47 (39.5)	500–999	34 (46.0)	200–499	4 (5.8)	85 (32.4)
	<750	4 (3.4)	<500	1 (1.3)	<200	0	5 (2.0)

Total		119 (100.0)		74 (100.0)		69 (100.0)	262 (100)

CDC classification (CD4%)	≥25	85 (75.2)	≥25	46 (62.2)	>25	57 (85.1)	188 (74.0)
	15–24	28 (24.8)	15–24	25 (33.8)	15–24	10 (14.9)	63 (24.8)
	<15	0	<15	3 (4.0)	<15	0	3 (1.2)

Total		113 (100)		74 (100.0)		67 (100.0)	254 (100)
